# Automated and virus variant-programmable surrogate test qualitatively compares to the gold standard SARS-CoV-2 neutralization assay

**DOI:** 10.1038/s44298-024-00083-9

**Published:** 2024-12-30

**Authors:** Danielle W. Ali, Maggie L. Bartlett, Christopher D. Heger, Francisco Ramirez, Linwood Johnson, Kevin L. Schully, Eric D. Laing, Wei Wang, Carol D. Weiss, Emilie Goguet, Christopher C. Broder, Stephanie A. Richard, Nusrat J. Epsi, Brian Agan, David Tribble, Mark P. Simons, Timothy H. Burgess, Edward Mitre, Simon Pollett, Darci R. Smith

**Affiliations:** 1 Microbiology and Immunology Department, Biological Defense Research Directorate, Naval Medical Research Command, Fort Detrick, Maryland, MD USA; 2 ProteinSimple, a Bio-Techne brand, San Jose, CA USA; 3 Austere Environments Consortium for Enhanced Sepsis Outcomes (ACESO), Biological Defense Research Directorate, Naval Medical Research Command, Fort Detrick, MD USA; 4 Department of Microbiology and Immunology, Uniformed Services University of the Health Sciences, Bethesda, MD USA; 5 Center for Biologics Evaluation and Research, U.S. Food and Drug Administration, Silver Spring, MD USA; 6 Henry M. Jackson Foundation for the Advancement of Military Medicine, Inc., Bethesda, MD USA; 7 Infectious Disease Clinical Research Program, Department of Preventive Medicine and Biostatistics, Uniformed Services University of the Health Sciences, Bethesda, MD USA

**Keywords:** SARS-CoV-2, Antibodies

## Abstract

The ongoing emergence of new severe acute respiratory syndrome coronavirus 2 (SARS-CoV-2) variants underscores the need for rapid, adaptable, high-throughput testing. However, assays for neutralizing antibodies, which are a good measure of viral protection, usually require cell culture and either infectious SARS-CoV-2 or pseudotyped viral particles. To circumvent the challenges of cell-based assays, SARS-CoV-2 surrogate virus neutralization tests (sVNTs) measure inhibition of the binding of the spike (S) protein receptor binding domain (RBD) to its receptor, human angiotensin-converting enzyme 2 (hACE2) by neutralizing antibodies. Here we tested a prototype automated microfluidic cartridge-based sVNT platform using SARS-CoV-2 wild-type (WT) and B.1.617.2 (Delta) variant RBDs. This sVNT showed a high correlation with cell-based neutralization assays for biospecimens collected post-COVID-19 vaccination and post-SARS-CoV-2 infection as well as for pre-pandemic SARS-CoV-2 negative sera. Thus, this assay, which takes less than 80 min, is a relatively simple, safe, and accurate alternative to traditional VNTs.

## Introduction

The emergence of SARS-CoV-2 viral variants and the potential for continued spread of the virus underscores the need for rapid, adaptable, surrogate neutralizing antibody screening assays suitable for biosafety level (BSL)-2 facilities. Correlates of protection are typically defined as the level of the immune response induced by the infection or the vaccine that provides protection against future infection, usually determined by neutralizing antibody titers quantified by neutralization assays^1^. Established methods include the plaque-reduction neutralization test (PRNT) and the virus microneutralization test (mVNT), which measure neutralizing antibodies that inhibit virus-mediated cytopathic effects^2^; however, both tests require live cells, infectious virus, BSL-3 containment, and days to complete. Although pseudovirus neutralization tests (pVNTs) that replace infectious virus with pseudotyped viral particles (such as those based on vesicular stomatitis virus or lentivirus) do not require BSL-3 containment, the procedure is laborious and also takes days to complete^3^. Surrogate virus neutralization tests (sVNTs), which require neither infectious virus or BSL-3 containment, measure antibody-mediated inhibition of the spike (S) protein receptor binding domain (RBD) binding to the human angiotensin-converting enzyme 2 (hACE2) receptor^4^. Current sVNTs eliminate the need for containment and are frequently ELISA or chemiluminescence-based^5^. Although not an exhaustive review of the literature, other sVNT may be based on the use of virus-like particles^6^ or beads coated with SARS-CoV-2 proteins^7^ and flow cytometric assays or a label-free biosensor-based approach^8^. Many of the sVNTs require multiple hands-on steps, long incubations, and equipment that is usually found only in a research or clinical laboratory.

We developed a high throughput, adaptable sVNT SARS-CoV-2 assay that is comparable to the gold standard cell-based mVNT and pVNT and that can be performed using the microfluidic cartridge-based, portable Ella platform^9^. As a proof of concept, we designed two variant-specific assays using the SARS-CoV-2 wild-type (WT) and B.1.617.2 (Delta) variant RBDs. We evaluated this assay using sera from multiple cohorts, including post-COVID-19 vaccination and post-SARS-CoV-2 infection biospecimens^10,11^, in addition to SARS-CoV-2 negative sera from pre-pandemic serum samples stored at the Department of Defense Serum Repository (DODSR) from individuals after infection with other endemic human coronaviruses (HCoVs) or influenza viruses^12^. This sVNT measured levels of neutralizing antibodies following vaccination or infection that were comparable to the traditional cell-based assays and the procedure required less than an 80 min run on a small and automated platform that does not require additional laboratory biosafety precautions.

## Materials and methods

### Curation of sera for assay calibration and evaluation of sensitivity and specificity

The SARS-CoV-2 IgG-negative panel (Supplemental Table 1) (*n* = 203) comprised pre-COVID-19 DODSR negative samples (*n* = 50) or samples with known HCoV or influenza virus infections (*n* = 50), and pre-COVID-19-vaccination and pre-SARS-CoV-2 infection sera from the Prospective Assessment of SARS-CoV-2 Seroconversion (PASS study) (*n* = 103)^10^. Briefly, PASS is a longitudinal cohort study of healthcare workers that includes the serial collection of serum samples and regular assessments of vaccination and infection events. Participants in the PASS study, who were documented to be seronegative with no prior COVID diagnosis upon study entry between August of 2020 and February of 2021, have been followed serially since that time^10^ (Supplemental Table 2). The DODSR samples were collected before 2020^12^ and a were PCR-confirmed to be associated with prior seasonal HCoV infections, H229E (*n* = 9), HKU1 (*n* = 9), NL63 (*n* = 10), or OC43 (*n* = 8), or influenza A (*n* = 6) or influenza B (*n* = 8).

We also curated a panel of sera obtained from individuals with prior SARS-CoV-2 infection and/or COVID-19 vaccination (Supplemental Table 1). Here we used post-vaccination sera from the PASS post-vaccination cohort (*n* = 103), which is the same cohort that provided pre-immune sera and had no prior SARS-CoV-2 infection (Supplemental Table 2)^10^. Additionally, we used post-infection sera from the Epidemiology, Immunology, and Clinical Characteristics of Emerging Infectious Diseases with Pandemic Potential (EPICC study), in which participants located at various military treatment facilities were enrolled after SARS-CoV-2 infection (*n* = 17 individuals) (Supplemental Tables 3 and 4)^13^. Serum samples were collected at enrollment (early, <60 days post-symptom onset), 6 months post-enrollment, and 12 months post-enrollment (*n* = 51 serum samples total).

#### Simple Plex^TM^ sVNT assay

**Fig. 1:**
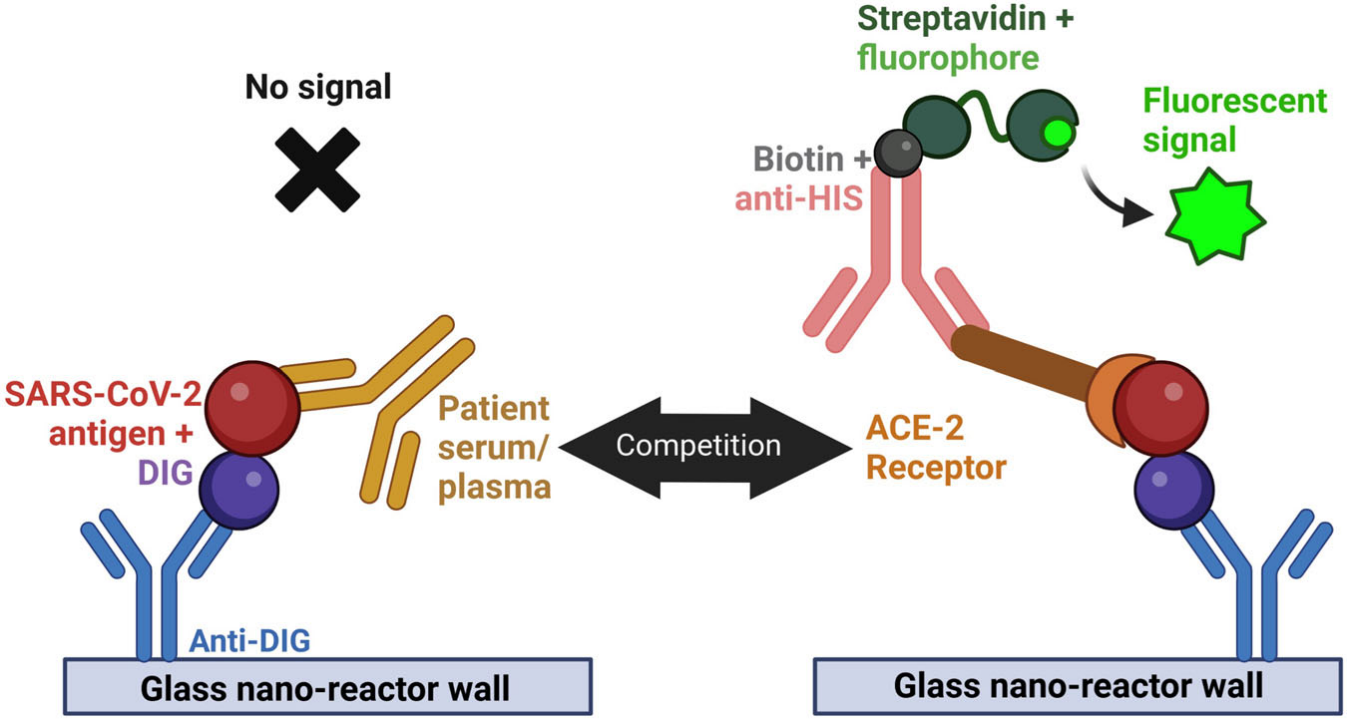
Schematic diagram of the sVNT for SARS-CoV-2. The capture reagent is a DIG-labeled RBD (red) that binds to the anti-DIG antibody attached to the GNR wall (blue). Capture-protein-specific antibodies (gold) in the serum compete with His-tagged ACE-2 (brown and orange) to decrease the signal. The ACE-2-His bound to the DIG-labeled RBD is measured using a biotinylated anti-His antibody (pink) that produces a fluorescent signal when the biotin binds to a fluorescent streptavidin conjugate (green).

The Simple Plex^TM^ sVNT assay is based on the binding of tethered Digoxigenin (DIG)-labeled RBD to a His-tagged ACE2 receptor (ACE2-His). The ACE2-His bound to the tethered DIG-labeled RBD was measured using a biotinylated anti-His antibody that produced a fluorescent signal when the biotin bound to a fluorescent streptavidin conjugate. Anti-RBD antibodies in serum competed with ACE2-His for binding to DIG-RBD, thereby decreasing the fluorescent signal (Fig. 1). Reagents for the detection of SARS-CoV-2 antibodies using Simple Plex^TM^ Digoxigenin Open Cartridges (ST01D-OT-006229, ProteinSimple, San Jose, CA) were described previously^14^. Following a modified procedure from the Simple Plex^TM^ Quick Start Guide, we conjugated WT S1-RBD (R&D Systems, Minneapolis, MN 10499-CV-100) and Delta B.1.617.2 S1-RBD (R&D Systems 10901-CV-100) with DIG. We determined RBD concentrations using a bicinchoninic acid (BCA) protein assay (Kit-23227, Pierce^TM^, Thermo Fisher Scientific Rockford, IL) and prepared the DIG-labeled stocks at 10 µg/mL in sample diluent 13 (SD13; part # 896069, ProteinSimple). We determined the half maximal effective concentration at 50% (EC_50_) of ACE2-His (R&D Systems 933-ZN) for the WT and Delta DIG-S1-RBDs using a Simple Plex^TM^ customizable 48-DIG open cartridge with a dilution series of ACE2-His and 10 μg/mL of each RBD, measuring the relative fluorescence unit (RFU) response with a biotinylated mouse anti-His-tag IgG antibody (R&D Systems BAM050) that was prepared at 3.33 µg/mL in SD13.

For the assay setup, the Simple Plex^TM^ open cartridge was pre-loaded with 10 μg/mL of each DIG-conjugated RBD resuspended in SD13 in duplicate. Serum samples were resuspended in SD19 (part # 992518, ProteinSimple) at a 1:5 dilution and then serially diluted 1:3 seven times. SD19 diluent was included as a negative control, and 0.3–30 µg/mL of the monoclonal antibody CR3022 (WRAIR EG-03-13–20, kindly provided by Dr. M. Gordon Joyce^15^) was the positive control, which we found to be weakly neutralizing. The CR3022 antibody was used to determine the IC_50_ values from four independent replicates, which were found to be consistent, with a mean IC_50_ of 1.3 × 10^−3 ^µM and a standard deviation of 1.6 × 10^−4 ^µM. The coefficient of variation was 12.23%, indicating moderate precision of the assay. In the 96-well sample preparation plate, each sample or control was mixed 1:1 with ACE2-His. Capture antigen and detector antibody were pipetted into their respective cartridge inlets, and then the combined ACE2-His and sample (or control) mixture was transferred from the sample preparation plate to the cartridge. The cartridge was loaded into the Ella Automated Immunoassay System (600-100, ProteinSimple) following the manufacturer’s instructions. Triplicate glass nano reactors (GNRs) coated with anti-DIG antibody captured the RBDs, and in ~80 min, the instrument measured individual RFU values from each GNR and calculated mean RFU values for the triplicate GNRs. RFU means were converted to a percent (%) inhibition value for each sample at every dilution point by the following formula: Inhibition [%] = [1 – (RFU mean/negative control)] × 100. We used analyses of the 50 SARS-CoV-2 negative sera samples provided by the DODSR and collected before the emergence of the virus to determine a percent inhibition threshold for assay positivity. We used analyses of pre-vaccination, post-vaccination, and post-infection negative sera (from the PASS and EPICC protocols) to determine receiver operating characteristic (ROC) curves.

#### pVNT and mVNT assay

Neutralizing antibody titers were determined by pVNT assays as described previously^16,17^. Briefly, heat-inactivated sera was serially diluted, mixed with S-pseudovirus, and incubated at 37 °C for 1 h. The resulting mixture was transferred in duplicate to a flat-bottom culture plate seeded with 293T-ACE2/TMPRSS2 cells and incubated at 37 °C for 48 h. Following incubation, the cells were lysed and firefly luciferase activity was read in a luminometer. Neutralizing titers were calculated as described previously^17^.

Neutralizing antibody titers were determined by mVNT assays as described previously^18^. Briefly, heat-inactivated sera was serially diluted, mixed with WT SARS-CoV-2 virus stock, and incubated at 37 °C for 1 h. The resulting mixture was added in triplicate to Vero 81 cells in a 96 well microtiter plate and incubated at 37 °C for ~84 h. The plates included negative controls for cells only (no virus), and positive controls with cells plus virus (no sera). Following incubation, the cells were washed, fixed, and an ELISA to detect SARS-CoV-2 S protein was performed. Cutoffs were determined using the mean value of nine virus only controls and neutralizing titers were calculated as described previously^18^.

The pVNT and mVNT assays were run on the PASS post-vaccination and EPICC post-infection sera (Supplemental Tables 2 and 3). Correlations between sVNT, pVNT and mVNT assay read-outs were performed using Spearman’s correlation.

### Calculations and statistics

Optimal cutoffs for sVNT assays were determined by generation of ROC curves (using data from the lowest dilution, 1:10) and calculation of the Youden index. Correlations between assays were determined by Spearman’s rank correlation. EC_50_ values between multiple assays were compared using Dunn’s test following normality testing. All statistical analyses were performed in GraphPad Prism 8.3.1 or SAS.

### Ethical standards

The PASS study protocol (IDCRP-126) was approved by the Uniformed Services University Institutional Review Board. The study was conducted in accordance with the local legislation and institutional requirements, and all participants provided written informed consent to participate in this study. The EPICC study (IDCRP-085) was approved by the Uniformed Services University Institutional Review Board and all study participants provided written consent when enrolled in the study. This observational cohort study in a convenience sample of MHS beneficiaries was conducted following good clinical practice and according to the Declaration of Helsinki guidelines. All human serum samples were de-identified, and the protocol for this study was approved by the Naval Medical Research Command’s Institutional Review Board in compliance with all applicable federal regulations governing the protection of human subjects.

## Results

### The sVNT has high sensitivity and specificity to WT and Delta RBDs

**Fig. 2:**
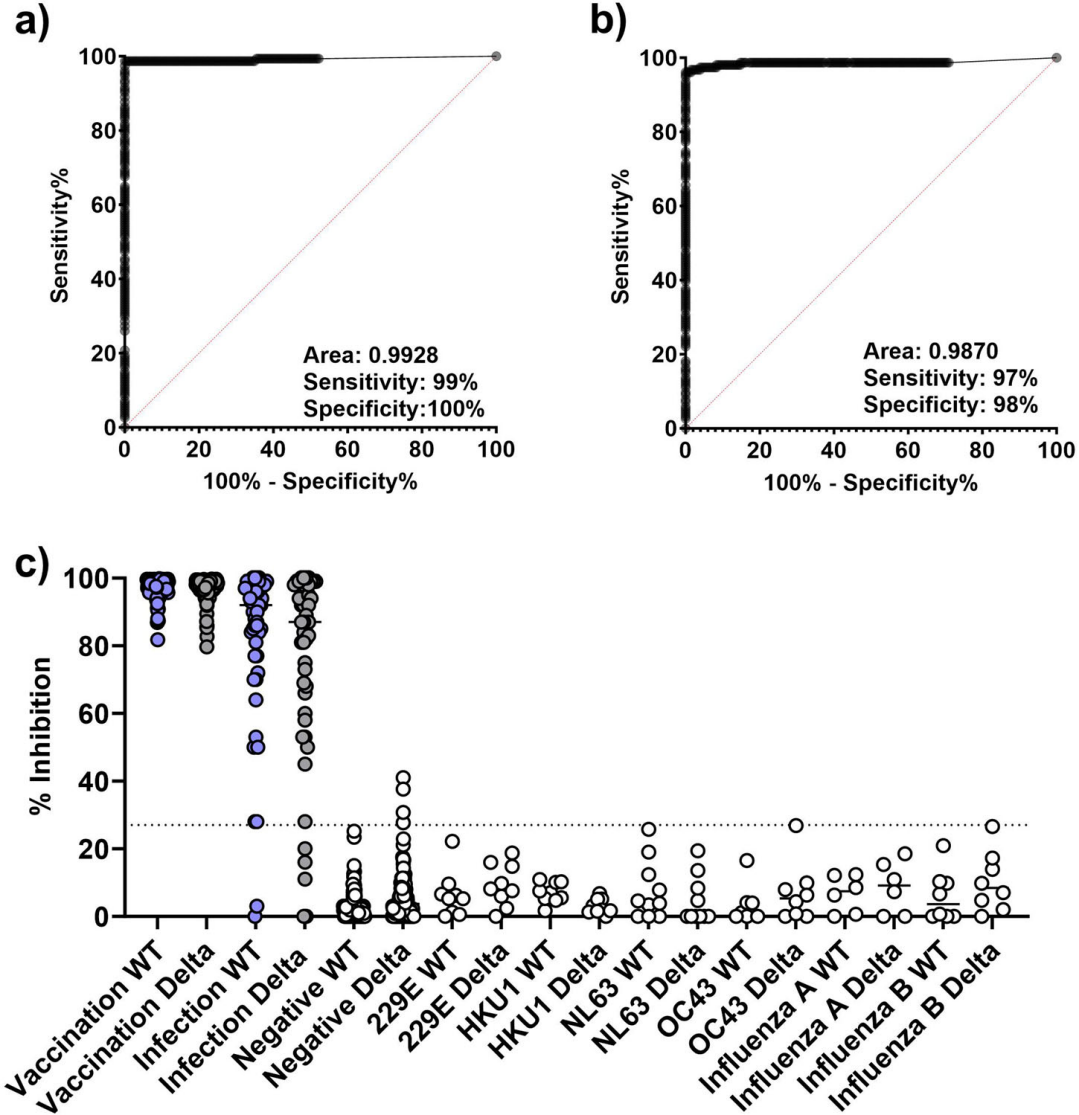
Determination of inhibition cutoff percent. ROC plot showing specificity and sensitivity for (**a**) WT and (**b**) Delta RBD. **c** The percent inhibition for post-vaccination/infection samples vs. WT-RBD (blue circles) or Delta-RBD (gray circles) and a panel of negative controls (white circles) comprised of pre-vaccination sera collected prior to 2019 and from other respiratory infections. The dotted line represents the 27% cutoff. Vaccination samples (*n* = 103) from the PASS cohort received the two-dose BNT162b2 (Pfizer) series, whereas the infection samples (*n* = 51 serum samples from 17 individuals at 3 time points) represent individuals positive for SARS-CoV-2 with varied vaccination status from the EPICC cohort. The negative control sample panel (*n* = 203) comprised pre-vaccination confirmed negative samples (*n* = 103), sera collected prior to the outbreak of SARS-CoV-2 (*n* = 50), and samples from other respiratory viruses and human coronaviruses (*n* = 50): 229E (*n* = 9), HKU1 (*n* = 9), NL63 (*n* = 10), OC43 (*n* = 8), influenza A (*n* = 6) and influenza B (*n* = 8).

We analyzed the WT RBD sVNT (WT-sVNT) and Delta RBD sVNT (Delta-sVNT) based on the percent inhibition calculated by the automated platform, and we produced ROC curves to determine assay performance, sensitivity, and specificity. We determined a cut-off of 27% inhibition would provide optimal sensitivity and specificity based on negative sample reactivities and the Youden index. As seen in Fig. 2a, b, this cut-off provides high sensitivity and specificity for both sVNT assays (WT-sVNT: AUC = 0.9928, sensitivity = 98%, specificity = 100%; Delta-sVNT: AUC = 0.9870, sensitivity = 97%, specificity = 96%). With an AUC of 0.9928 and 0.9870 for the WT-sVNT and Delta-sVNT, respectively, both assays demonstrate an excellent diagnostic accuracy, meaning it effectively discriminates between positive and negative samples. Both sVNT assays were positive (>27% inhibition) for all post-vaccination samples tested, and for >90% of post-infection samples tested (Fig. 2c). With regards to specificity, 100% of the negative serum samples were below the cut-off of 27% for WT-sVNT and 97.4% were below the cut-off for Delta-sVNT. All the serum samples from individuals with HCoVs and influenza virus infections were below our 27% cut-off, demonstrating that this sVNT can distinguish between neutralizing antibodies to SARS-CoV-2, other HCoVs, and influenza virus with little or no cross-reactivity.

### WT-sVNT is qualitatively comparable to the gold standard mVNT and cell-based neutralization assays, but correlations vary by post-infection timepoint

**Fig. 3:**
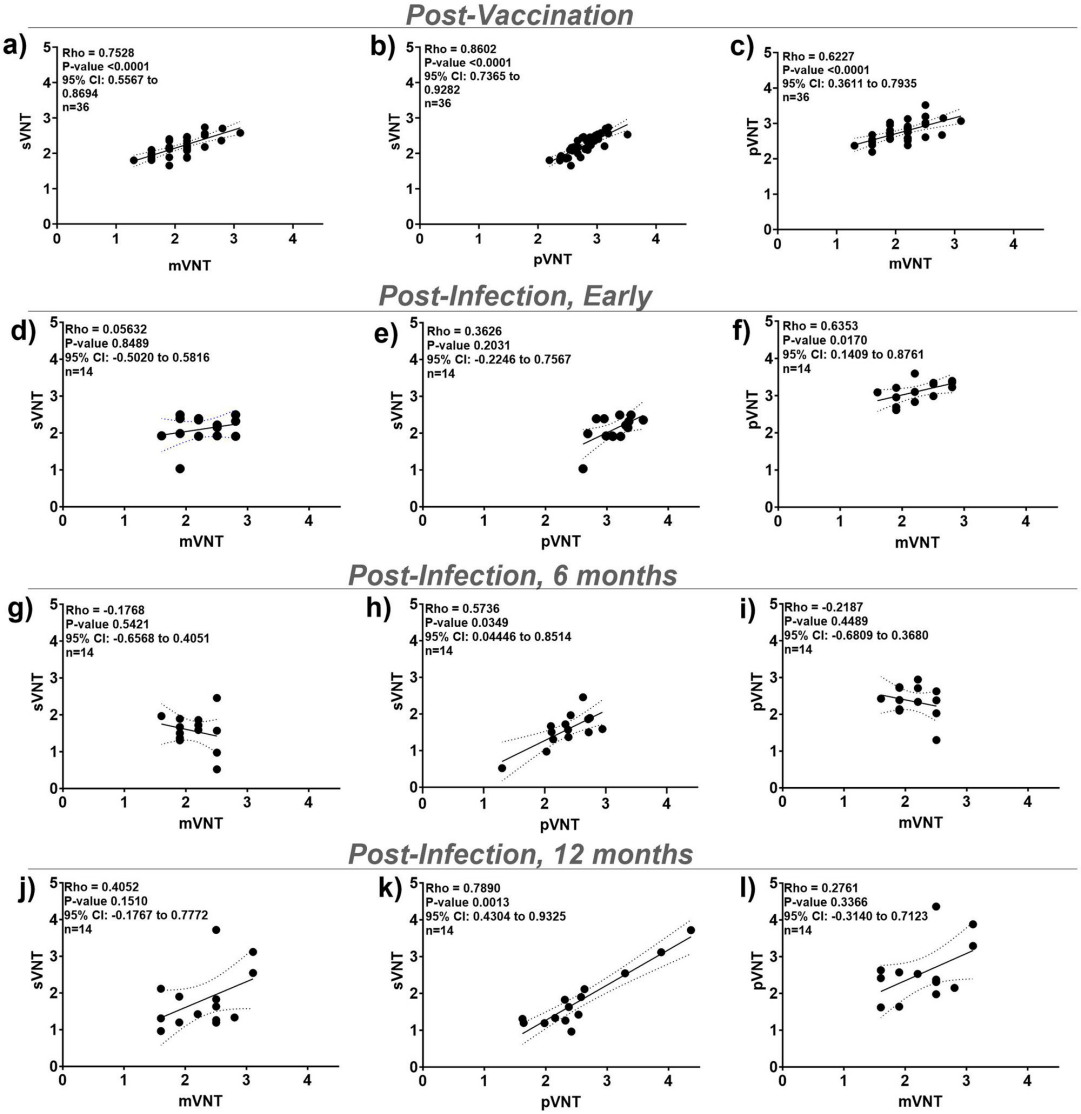
Correlations for the sVNT, mVNT, and pVNT antibody titers. (log EC_50_). Correlations for sVNT, mVNT, and pVNT antibody titers (log EC_50_) against WT RBD. **a**–**c** Post-vaccination (*n* = 36), (**d**–**f**) early post-infection (*n* = 14), (**g**–**i**) 6 months post-infection (*n* = 14), and (**j**–**l**) 12 months post-infection (*n* = 14). The correlation was determined by Spearman’s rho. Linear regression line (solid line), Spearman’s 95% confidence interval (CI) (dashed line), Spearman’s *P*-value, and *n* are shown.

We compared our sVNT assays to the cell-based mVNT and pVNT (Table 1) with 36 of the post-vaccination serum samples from the PASS study and 14 post-infection serum samples per time-point (early, 6 months, and 12 months) from the EPICC study. Three post-infection serum samples per time-point from the EPICC study were not included because no EC_50_ could be calculated due to low or no antibodies detected in those samples. Spearman’s test showed strong correlations between neutralizing antibody titers for sVNT versus the cell-based mVNT and pVNT for post-vaccination (*n* = 36) sera (the sVNT vs the mVNT ρ = 0.75, *P* < 0.0001, 95% CI = 0.55 to 0.87; sVNT vs pVNT ρ = 0.86, *P* < 0.0001, 95% CI = 0.73 to 0.93) (Fig. 3a–c). However, there was variability in the strength of the correlation of the post-infection titers between the sVNT and traditional cell-based assays. Early post-infection, there was a weak correlation between the sVNT and the mVNT (ρ = 0.05, *P* = 0.85), whereas the correlation between the sVNT and the pVNT was moderate (ρ = 0.36, *P* = 0.20) (Fig. 3d–2f). At 6 months post-infection, there was poor correlation between the sVNT and the mVNT (ρ = − 0.17, *P* = 0.54) but a moderate correlation between the sVNT and the pVNT (ρ = 0.57, *P* = 0.03) (Fig. 3g–2I). Finally, at 12 months post-infection, there was a moderate correlation between the sVNT and the mVNT (ρ = 0.40, *P* = 0.15) and a strong correlation between the sVNT and the pVNT (ρ = 0.79, *P* = 0.0013) (Fig. 3J–2L).

**Table 1:** Comparison of neutralization assays

Assay	Acronym	Mechanism	Time	Safety	Performance	Caveat
Plaque Reduction Neutralization Test	PRNT	Infectious Virus/Cell Based	5 days	BSL-3	Gold standard	Time/specialized containment
Microneutralization Assay	mVNT	Infectious Virus/Cell Based	2–4 days	BSL-3	Comparable to PRNT	Time/specialized containment
Pseudovirus Neutralization Assay	pVNT	Lentivirus, VSV, or other BSL-2 virus /Cell Based	2–3 days	BSL-2	Comparable to PRNT	Time/not the whole virus
Surrogate Virus Neutralization Test	sVNT	Molecular interaction-based/e.g. ELISA, flow cytometry, bead, or sensor based	1–4 h	BSL-1 or 2	Comparable to PRNT	Specialized equipment, not the whole virus

### Consistency of titers across assays and cohorts

**Fig. 4:**
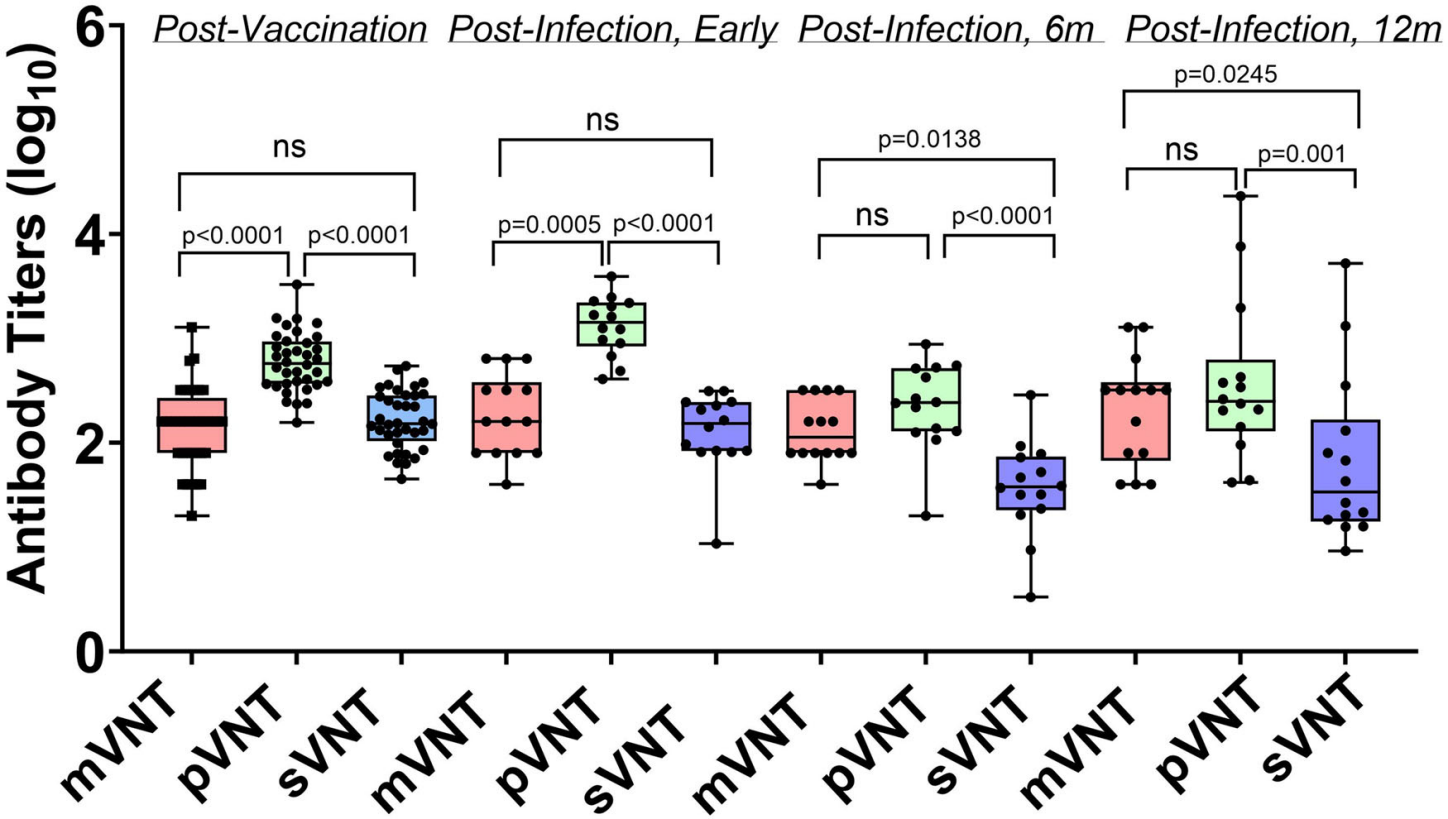
Comparison of titers of antibodies against WT RBD for the mVNT, pVNT, and sVNT. Differences between mean log EC_50_ titers post-vaccination (*n* = 36), early post-infection (*n* = 14), 6 months post-infection (*n* = 14), and 12 months post-infection (*n* = 14). The upper and lower bars represent the minimum/maximum, ns = not significant (*p* > 0.05). Dunn’s statistical significance following normality testing was calculated using GraphPad

In order to provide context for interpreting the assay correlation results, we compared neutralizing antibody titers for serum samples from the post-vaccination and post-infection groups for the mVNT, the pVNT, and the sVNT (Fig. 4). We observed a range of titers and found no significant differences between the sVNT and the mVNT for the samples from the post-vaccination and early post-infection groups. The 6-month post-infection and 12 month post-infection samples showed weakly significant differences (*P* = 0.0138 and *P* = 0.0245, respectively) in titer between the sVNT and the mVNT. There were also differences between the antibody titers for the sVNT vs the pVNT for all groups of samples; however, these are unlikely to have clinical significance since all samples had a relatively high neutralizing antibody titer. These differences also likely reflect the variability in the dilution schemes for the different assays which were run in independent labs.

### sVNT titers for WT and delta RBD

**Fig. 5:**
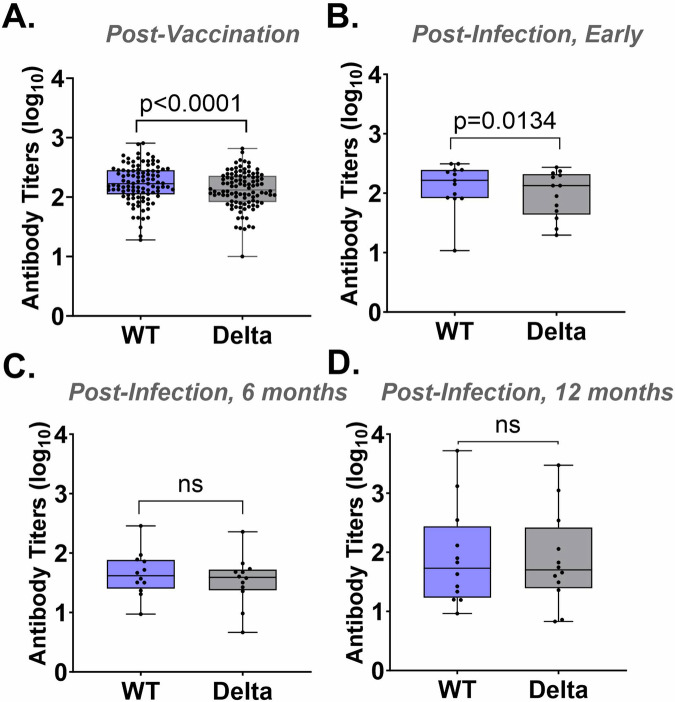
Comparison of antibody titers using the WT-sVNT and the Delta-sVNT. Log EC_50_ antibody titers for WT-sVNT and Delta-sVNT for samples (**A**) post-vaccination (*n* = 102), (**B**) early post-infection, (*n* = 13), (**C**) 6 months post-infection (n = 12), and (**D**) 12 months post-infection (*n* = 12). The upper and lower bars represent the minimum/maximum, ns = not significant (*p* > 0.05). Wilcoxon statistical significance was calculated using GraphPad.

To determine whether the sVNT can be used to detect different SARS-CoV-2 variants, we directly compared serological measurements by WT-sVNT vs. Delta-sVNT. The mean antibody titer was significantly lower for the Delta-sVNT compared to the WT-sVNT in the post-vaccination (*P* < 0.0001) and early post-infection serum samples (*P* = 0.0134) but not for the 6 month post-infection and 12 month post-infection samples (Fig. 5A–D).

## Discussion

Testing for SARS-CoV-2 infection and immunity remains a high priority as new variants emerge. Neutralizing antibody titers that correlate with SARS-CoV-2 infection can provide information on thresholds for protective immunity^19–21^. However, traditional cell-based neutralization assays are slow and expensive. In this study, we developed a sensitive and specific sVNT that runs in under 80 min on an automated immunoassay platform designed by Simple Plex^TM^. We evaluated its performance compared to the gold standard cell-based mVNT and pVNT. This sVNT assay demonstrated high sensitivity and specificity for both WT-sVNT and Delta-sVNT and is easily customized for new variants. Similar to other sVNTs, our sVNT had an inhibition cut-off of 27%^22–28^. Both the pVNT and the mVNT showed moderate to strong correlation with the sVNT for post-vaccination serum samples and a strong correlation for early and 12 months post-infection samples, but not for samples collected 6 months after infection. Although the lower neutralizing antibody response to RBD after infection versus after vaccination resulted in better test performance for post-vaccination samples, overall, the data indicate that our sVNT qualitatively identified anti-RBD antibodies after vaccination and after infection.

While the sVNT has been shown to be suitable to detect neutralizing antibodies in our study and by others^22–28^, a known limitation is that the assay only detects RBD-targeting neutralizing antibodies and will more accurately quantify neutralizing antibodies in vaccinees compared to convalescent individuals. The sVNT likely performed better with the post-vaccination samples compared to the post-infection samples because the immune response to vaccination is specific for producing antibodies to the spike protein, which includes RBD-targeting neutralizing antibodies. The immune response after natural infection generates neutralizing antibodies that may target other epitopes on the SARS-CoV-2 virion that are not detected by the sVNT. However, the RBD-targeting neutralizing antibodies have been demonstrated to be immunodominant during SARS-CoV-2 infection^29^. Although our assay does not capture the spectrum of immune responses to SARS-CoV-2 infection, it measures reactivities to RBD comparable to canonical VNTs.

Given the high mutation rates in SARS-CoV-2 with the ongoing emergence of variants, it is important that assays can be adapted for variants of concern (VOC)^30^. With this automated sVNT, it is feasible to change the target variants rapidly as they emerge. Although this platform is adaptable for new variants, whether it will be necessary to create a new RBD-based bioreagent will likely depend on the specific variant^31^. Because all our samples were collected during the same phase of the pandemic, we will need to assess a wider range of samples to identify shifts in epitope recognition, including re-evaluation of PRNT and sVNT correlation across newer variants. Additionally, because the longitudinal post-infection cohort was small (n = 51 sera from 17 individuals), we need future studies that examine larger sample sizes.

Reproducible and scalable serology assays that can be adapted to evaluate the immune response to emerging pathogens are critical for timely responses and interventions against diseases^32^. In summary, our analysis findings here indicate good performance of the Simple Plex^TM^ microfluidic immunoassay for post-vaccine immunity measurement, but varying performance of quantitative measurement from post-infection sera, when compared to reference assays. These findings prompt further assay target validations on prospective independent sera sets collected from those infected and/or vaccinated against historical and emerging variants.

## Supplementary information

Supplementary material

## Data Availability

The datasets generated and/or analyzed during the current study available from the corresponding author on reasonable request.

## References

[1] Krammer, F. A correlate of protection for SARS-CoV-2 vaccines is urgently needed. *Nature Medicine* **27**, 1147–1148 (2021). 34239135 10.1038/s41591-021-01432-4

[2] Amanat, F. et al. An in vitro microneutralization assay for SARS-CoV-2 serology and drug screening. *Curr. Protoc. Microbiol.* **58**, e108, 10.1002/cpmc.108 (2020). 32585083 10.1002/cpmc.108 PMC7361222

[3] Nie, J. et al. Establishment and validation of a pseudovirus neutralization assay for SARS-CoV-2. *Emerg. Microbes Infect.* **9**, 680–686 (2020). 32207377 10.1080/22221751.2020.1743767 PMC7144318

[4] Lu, Y. et al. Advances in neutralization assays for SARS-CoV-2. *Scandinav. J. Immunol.* **94**, e13088, 10.1111/sji.13088 (2021).

[5] Pieri, M. et al. Performance evaluation of four surrogate virus neutralization tests (sVNTs) in comparison to the in vivo gold standard test. *Front Biosci*. **27**, 74 (2022). 10.31083/j.fbl2702074 35227017

[6] Jaron, M. et al. Baculovirus-free SARS-CoV-2 virus-like particle production in insect cells for rapid neutralization assessment. *Viruses* **14**, 2087 (2022). 10.3390/v14102087 PMC9606917 36298643

[7] Yao, X. et al. A highly sensitive bead-based flow cytometric competitive binding assay to detect SARS-CoV-2 neutralizing antibody activity. *Front Immunol.* **13**, 1041860 (2022). 36532082 10.3389/fimmu.2022.1041860 PMC9748424

[8] Luo, Y. R., Yun, C., Chakraborty, I., Wu, A. H. B. & Lynch, K. L. A SARS-CoV-2 label-free surrogate virus neutralization test and a longitudinal study of antibody characteristics in COVID-19 patients. *J. Clin. Microbiol.* **59**, e0019321 (2021). 33827900 10.1128/JCM.00193-21 PMC8218741

[9] Aldo, P., Marusov, G., Svancara, D., David, J. & Mor, G. Simple Plex(™): a novel multi-analyte, automated microfluidic immunoassay platform for the detection of human and mouse cytokines and chemokines. *Am. J. Reprod Immunol.* **75**, 678–693 (2016). 27170460 10.1111/aji.12512 PMC5084752

[10] Jackson-Thompson, B. M. et al. Prospective assessment of SARS-CoV-2 seroconversion (PASS) study: an observational cohort study of SARS-CoV-2 infection and vaccination in healthcare workers. *BMC Infectious Dis.* **21**, 544 (2021). 10.1186/s12879-021-06233-1 PMC8188741 34107889

[11] Shenoy, A., Marwaha, P. K. & Worku, D. A. CD8 encephalitis in HIV: a review of this emerging entity. *J. Clin. Med.* **12**, 770 (2023). 10.3390/jcm12030770 PMC9917721 36769419

[12] Rubertone, M. V. & Brundage, J. F. The defense medical surveillance system and the department of defense serum repository: glimpses of the future of public health surveillance. *Am. J. Public Health* **92**, 1900–1904 (2002). 12453804 10.2105/ajph.92.12.1900 PMC1447349

[13] Song, G. et al. Cross-reactive serum and memory B-cell responses to spike protein in SARS-CoV-2 and endemic coronavirus infection. *Nat. Commun.* **12**, 2938 (2021). 34011939 10.1038/s41467-021-23074-3 PMC8134462

[14] Johnson, L., Bartlett, M. L., Ramirez, F., Heger, C. D. & Smith, D., D. *Development of Automated Microfluidic Immunoassays for the Detection of Sars-Cov-2 Antibodies and Antigen.* https://ssrn.com/abstract=4567918 (2023). 10.1016/j.jim.2023.113586 38040191

[15] Sankhala, R. S. et al. Antibody targeting of conserved sites of vulnerability on the SARS-CoV-2 spike receptor-binding domain. *Structure* **32**, 131–147.e137 (2024). 38157856 10.1016/j.str.2023.11.015 PMC11145656

[16] Neerukonda, S. N. et al. Establishment of a well-characterized SARS-CoV-2 lentiviral pseudovirus neutralization assay using 293T cells with stable expression of ACE2 and TMPRSS2. *PLoS One* **16**, e0248348 (2021). 33690649 10.1371/journal.pone.0248348 PMC7946320

[17] Neerukonda, S. N., Vassell, R., Weiss, C. D. & Wang, W. Measuring neutralizing antibodies to SARS-CoV-2 using lentiviral spike-pseudoviruses. *Methods Mol. Biol.* **2452**, 305–314 (2022). 35554914 10.1007/978-1-0716-2111-0_18

[18] Laing, E. D. et al. SARS-CoV-2 antibodies remain detectable 12 months after infection and antibody magnitude is associated with age and COVID-19 severity. *medRxiv*10.1101/2021.04.27.21256207 (2021).

[19] Khoury, D. S. et al. Correlates of protection, thresholds of protection, and immunobridging among persons with SARS-CoV-2 infection. *Emerg Infect. Dis.* **29**, 381–388 (2023). 36692375 10.3201/eid2902.221422 PMC9881762

[20] Regev-Yochay, G. et al. Correlates of protection against COVID-19 infection and intensity of symptomatic disease in vaccinated individuals exposed to SARS-CoV-2 in households in Israel (ICoFS): a prospective cohort study. *Lancet Microbe.* **4**, e309–e318 (2023). 36963419 10.1016/S2666-5247(23)00012-5 PMC10030121

[21] Gilbert, P. B. et al. A Covid-19 milestone attained—a correlate of protection for vaccines. *N Engl. J. Med.* **387**, 2203–2206 (2022). 36507702 10.1056/NEJMp2211314

[22] Tan, C. W. et al. A SARS-CoV-2 surrogate virus neutralization test based on antibody-mediated blockage of ACE2–spike protein–protein interaction. *Nat. Biotechnol.* **38**, 1073–1078 (2020). 32704169 10.1038/s41587-020-0631-z

[23] Meyer, B. et al. Validation and clinical evaluation of a SARS-CoV-2 surrogate virus neutralisation test (sVNT). *Emerg. Microbes Infect.* **9**, 2394–2403 (2020). 33043818 10.1080/22221751.2020.1835448 PMC7605318

[24] Abe, K. T. et al. A simple protein-based surrogate neutralization assay for SARS-CoV-2. *JCI Insight* **5**, e142362 (2020). 10.1172/jci.insight.142362 PMC7566699 32870820

[25] Bosnjak, B. et al. Low serum neutralizing anti-SARS-CoV-2 S antibody levels in mildly affected COVID-19 convalescent patients revealed by two different detection methods. *Cell Mol. Immunol.* **18**, 936–944 (2021). 33139905 10.1038/s41423-020-00573-9 PMC7604543

[26] Marien, J. et al. Evaluation of a surrogate virus neutralization test for high-throughput serosurveillance of SARS-CoV-2. *J. Virol. Methods* **297**, 114228 (2021). 34224754 10.1016/j.jviromet.2021.114228 PMC8253660

[27] Perera, R. et al. Evaluation of a SARS-CoV-2 surrogate virus neutralization Test for Detection of antibody in human, canine, cat, and hamster sera. *J. Clin. Microbiol.* **59**, e02504-20 (2021). 10.1128/JCM.02504-20 PMC8111130 33139421

[28] Sekirov, I. et al. Performance comparison of micro-neutralization assays based on surrogate SARS-CoV-2 and WT SARS-CoV-2 in assessing virus-neutralizing capacity of anti-SARS-CoV-2 antibodies. *Access Microbiol.* **3**, 000257 (2021). 34888485 10.1099/acmi.0.000257 PMC8650845

[29] Premkumar, L. et al. The receptor binding domain of the viral spike protein is an immunodominant and highly specific target of antibodies in SARS-CoV-2 patients. *Sci. Immunol.* **5**, eabc8413 (2020). 10.1126/sciimmunol.abc8413 PMC7292505 32527802

[30] Markov, P. V. et al. The evolution of SARS-CoV-2. *Nat. Rev. Microbiol.* **21**, 361–379 (2023). 37020110 10.1038/s41579-023-00878-2

[31] Tian, Y. et al. Sensitivity and specificity of SARS-CoV-2 S1 subunit in COVID-19 serology assays. *Cell Discov.* **6**, 75 (2020). 33133635 10.1038/s41421-020-00224-3 PMC7589479

[32] Plotkin, S. A. Recent updates on correlates of vaccine-induced protection. *Front. Immunol.* **13**, 1081107 (2023). 10.3389/fimmu.2022.1081107 PMC9912984 36776392

