# Supplementary material for: Automated and virus variant-programmable surrogate test qualitatively compares to the gold standard SARS-CoV-2 neutralization assay
Source: Npj Viruses. 2024 Dec 30;2:68. doi: 10.1038/s44298-024-00083-9 (PMC11721378; doi:10.1038/s44298-024-00083-9)
Supplement: Supplementary file 1 — Supplementary material [file 44298_2024_83_MOESM1_ESM.pdf]

Supplemental Tables

Supplemental Table 1. SARS-CoV-2 IgG Negative and Positive Serum Panel

Sample type	Panel	Purpose	Source	Serum samples (n)	References
Pre-COVID-19 negative sera	Negative	Determine percent inhibition cutoff for assay positivity	DODSR[1]	50	12
Pre-COVID-19 seasonal human coronavirus or influenza virus infections	Negative	Determine specificity	DODSR[1]	50 [H229E (n = 9), HKU1 (n = 9), NL63 (n = 10), or OC43 (n = 8), or influenza A (n = 6) or influenza B (n = 8)]	12
Pre-COVID-19 vaccination and pre-SARS-CoV-2 infection	Negative	Determine ROC curves	PASS[2] study	103	10
Post-COVID-19 vaccination with	Positive	Determine ROC curves	PASS[2] study	103	10
Post-SARS-CoV-2 infection	Positive	Determine ROC curves	EPICC[3] study	51 (17 individuals at 3 time points)	13

[1]Department of Defense Serum Repository

[2]Prospective Assessment of SARS-CoV-2 Seroconversion (PASS study)

[3]Epidemiology, Immunology, and Clinical Characteristics of Emerging Infectious Diseases with Pandemic Potential (EPICC study)

Supplemental Table 2. Characteristics and demographics of the PASS study serum samples

	**N=103**
**Age group**	
18-44	55 (53.4%)
45-64	46 (44.7%)
65+	2 (1.9%)
**Sex**	
Female	70 (68.0%)
Male	33 (32.0%)
**Race**	
White	81 (78.6%)

Black	10 (9.7%)
Asian	10 (9.7%)
Others	2 (1.9%)
**Ethnicity**	
Non-Hispanic	102 (99.0%)
Hispanic	1 (1.0%)
**Charlson Comorbidity Index (CCI)**	
0	86 (83.5%)
1	9 (8.7%)
2	6 (5.9%)
3+	2 (1.9%)
**BMI category**	
Under/normal weight	43 (41.7%)
Overweight	39 (37.9%)
Obese	18 (17.5%)
Severely obese	3 (2.9%)
**Days between visit 1 and first vaccine dose**	
Median (Q1, Q3)	40 (34.0, 49.0)
**Days between second vaccine dose and visit 2**	
Median (Q1, Q3)	32.0 (29.0, 38.0)

Visit 1: pre-COVID-19 vaccination and pre-SARS-CoV-2 infection
Visit 2: post-COVID-19 vaccination with 2 vaccine doses

Supplemental Table 3. Characteristics of the EPICC study serum samples. The SARS-CoV-2 infections were determined to be ancestral variants.

	Early (N=17)	6m (N=17)	12m (N=17)
**Days post-symptom onset**			
Median (Q1, Q3)	35.0 (32.0, 41.0)	185.0 (177.0, 189.0)	356.0 (352.0, 360.0)
Min - Max	28.0 - 99.0	47.0 - 202.0	344.0 - 378.0
**Days post first positive**			
Median (Q1, Q3)	29.0 (27.8, 30.5)	179.0 (173.5, 182.2)	348.5 (344.8, 354.0)
Min - Max	24.0 - 93.0	46.0 - 197.0	338.0 - 372.0
**SARS-CoV-2 MFI**			

	Early (N=17)	6m (N=17)	12m (N=17)
Median (Q1, Q3)	29644.0 (28616.0, 30112.0)	27190.0 (26208.0, 27938.0)	24229.0 (16842.0, 30287.0)
Min - Max	26685.0 - 30833.0	11425.0 - 30622.0	3759.0 - 31044.0
**Days since first vaccine dose**			
0	0	0	1 (16.7%)
2	0	0	1 (16.7%)
6	0	0	2 (33.3%)
7	0	0	1 (16.7%)
42	0	0	1 (16.7%)
**Received first vaccine dose on or before date sample collected**			
0	17 (100.0%)	17 (100.0%)	11 (64.7%)
1	0 (0.0%)	0 (0.0%)	6 (35.3%)
**Days since second vaccine dose**			
4	0	0	1 (100.0%)
**Received second vaccine dose on or before date sample collected**			
0	17 (100.0%)	17 (100.0%)	16 (94.1%)
1	0 (0.0%)	0 (0.0%)	1 (5.9%)

Supplemental Table 4. Demographics of the EPICC study samples.

	N (N=17)
**Age group**	
18-44	3 (17.6%)
45-64	13 (76.5%)
65+	1 (5.9%)
**Sex**	
Male	9 (52.9%)
Female	8 (47.1%)
**Race/ethnicity**	

	N (N=17)
Asian or Pacific Islander	2 (11.8%)
Black	6 (35.3%)
Hispanic or Latino	6 (35.3%)
Other	1 (5.9%)
White	2 (11.8%)
**Charlson Comorbidity Index**	
0	6 (35.3%)
1-2	8 (47.1%)
3-4	2 (11.8%)
5+	1 (5.9%)
**Hospitalized**	
0	9 (52.9%)
1	8 (47.1%)
**BMI category**	
Under/normal weight	1 (5.9%)
Overweight	6 (35.3%)
Obese	4 (23.5%)
Severely obese	6 (35.3%)
**COVID Variant period**	
	1 (5.9%)
Ancestral	16 (94.1%)
**COVID Pangolin**	
	11 (64.7%)
B.1	2 (11.8%)
B.1.1.168	1 (5.9%)
B.1.333, B.1.369	1 (5.9%)
Unassigned	2 (11.8%)
